# Diversity of *Anaplasma phagocytophilum* Strains, USA

**DOI:** 10.3201/eid1506.081610

**Published:** 2009-06

**Authors:** Eric Morissette, Robert F. Massung, Janet E. Foley, A. Rick Alleman, Patrick Foley, Anthony F. Barbet

**Affiliations:** University of Florida, Gainesville, Florida, USA (E. Morissette, A.R. Alleman, A.F. Barbet); Centers for Disease Control and Prevention, Atlanta, Georgia, USA (R.F. Massung); University of California, Davis, California, USA (J.E. Foley); California State University, Sacramento, California, USA (P. Foley )

**Keywords:** Anaplasma, strain diversity, msp2/p44, OMPs, rickettsia, zoonoses, USA, dispatch

## Abstract

We analyzed the structure of the expression site encoding the immunoprotective protein MSP2/P44 from multiple *Anaplasma phagocytophilum* strains in the United States. The sequence of *p44ESup1* had diverged in *Ap-variant 1* strains infecting ruminants. In contrast, no differences were detected between *A. phagocytophilum* strains infecting humans and domestic dogs.

*Anaplasma phagocytophilum* (order Rickettsiales) has a broad host range and infects humans as well as numerous other animal species (*1*). It has been known as a ruminant pathogen in Europe since at least 1932 but in recent years has emerged as a cause of disease in humans in the United States and Europe. The number of cases reported to the Centers for Disease Control and Prevention has increased from 537 in 2004 to 834 in 2007. Similarly, the number of dogs with clinical anaplasmosis has apparently increased. Strains clearly differ; not all appear to be capable of infecting humans or mice (*2*) or to cause persistent infections. These differences have stimulated the search for molecular markers of strain phenotypes and host tropisms. Although much strain variation has been identified, these markers have not been clearly linked to host tropisms except for 16S rRNA and the US *Ap-variant 1* (*Ap-V1*). *Ap-V1* differs from a human strain (*Ap-ha*) by a 2-bp substitution in the 16S rRNA sequence (*3*) and appears to be restricted to ruminant species (*2*,*4*). Because a 2-bp difference in 16S rRNA is minimally informative, we selected the *msp2/p44* expression site to investigate other potential differences between *Ap-V1* and other strains. In the related organism, *A. marginale*, the genomic repertoire of *msp2* pseudogenes has been associated with the ability, or lack of ability, of strains to superinfect and cause epidemic spread of the organism (*5*).

## The Study

Different isolates of the *Ap-V1* strain were obtained from Rhode Island and Minnesota. Genomic DNA was extracted from infected goat blood, infected *Ixodes scapularis* ticks, and cell culture–grown isolates (ISE6) as described by Massung et al. (*6*). Other genomic DNA was isolated as described previously (*7*) from whole infected blood or HL-60 cultures. Dog blood was obtained from naturally infected animals identified by private practitioners in New York and Minnesota. Genomic DNA from the horse MRK strain of *A. phagocytophilum* (*8*) was isolated from infected equine neutrophils.

PCR amplification, sequencing, and analysis of the *msp2/p44* expression site were performed by methods described previously (*7*). We used oligonucleotide primers AB1207 (5′-GGGAGTGCTCTGGTTAGATTTAGG-3′) and AB1221 (5′-ATAGAACAAGAGCAGGGAGAAGAC-3′) or AB1207 and AB1058 (5′-GAACCATCCCCTTAAAATACTTTC-3′) to amplify the *msp2/p44* gene, the upstream gene *p44ESup1,* and the intergenic region between them. To amplify just the *msp2/p44* gene in the expression site and to determine hypervariable region sequences, we used AB1221 and AB1266 (5′-GAAGAAGAGATTGGACTTTTGATCTGTC-3′) or AB1221 and AB1267 (5′-GAGGAAGAGATTGGACTTTTGAGCTGTC-3′). The sequences determined here have been assigned GenBank accession nos. FJ467331–FJ467340.

**Figure 1:**
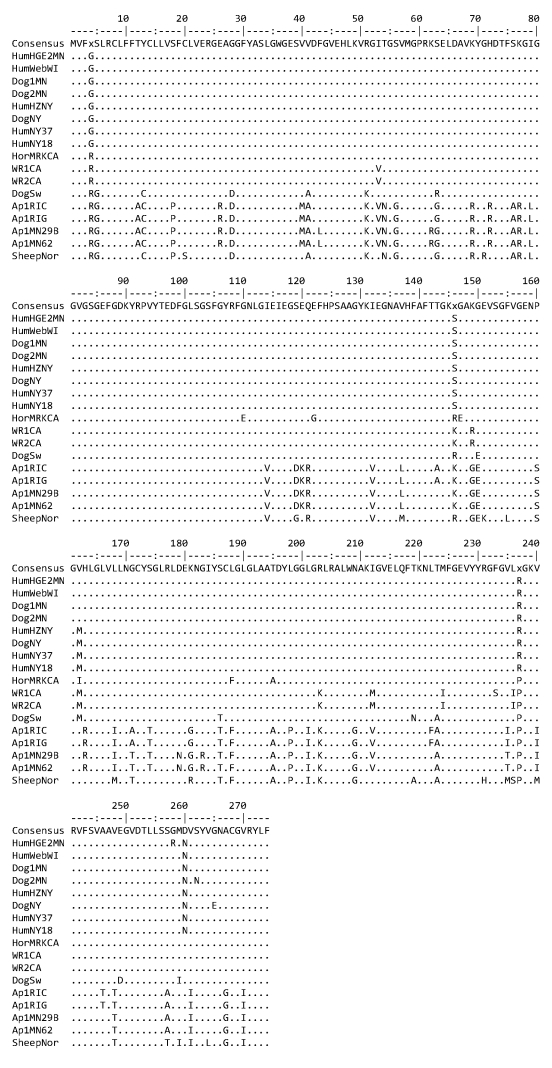
Diversity in the amino acid sequences encoded by *p44ESup1/omp-1n* in US and European strains of *Anaplasma phagocytophilum*. All strains are from the United States (the state is indicated in the strain designation) except for the strain from the sheep from Norway (SheepNor) and the dog from Sweden (DogSw). Human-origin strains are HZNY, NY18, NY37, WebWI, and HGE2MN; dog strains are Dog1MN, Dog2MN, and DogNY; wood rat (*Neotoma fuscipes*) strains are WR1CA and WR2CA; the horse strain is HorMRKCA; *Ap-V1* strains are Ap1RIC (culture derived), Ap1RIG (isolated from an infected goat), Ap1MN29B, and Ap1MN62 (both Ap1MN strains were derived from naturally infected *Ixodes scapularis* ticks). Sequences were from either this study or GenBank: accession nos. DQ519565 (SheepNor), DQ519566 (DogSw), CP000235 (HZ), AY164490 (NY18), AY137510 (NY37), AY164491 (Webster), and AY164492 (HGE2).

The expression site encoding MSP2/P44 is composed of the MSP2/P44 coding region itself, including a central hypervariable region, an intergenic region containing binding sites for a regulatory transcription factor ApxR, and an upstream gene known either as *p44ESup1* or *omp-1n* (*9*,*10*). In our study, the *p44ESup1* gene appeared to be experiencing purifying or stabilizing selection. Evolutionary analysis using MEGA4 (*11*; Nei-Gojobori method with the Jukes-Cantor correction) showed a low ratio of nonsynonymous-to-synonymous substitutions (mean dN 0.053, dS 0.296; dN/dS ratio 0.179). The *Ap-V1* strains from Rhode Island and Minnesota shared many substitutions with a sheep strain from Norway that were not present in the other *A. phagocytophilum* strains (Figure 1). The *p44ESup1*gene in strains isolated from 5 persons from Wisconsin, Minnesota, and New York most closely resembled *p44ESup1* in strains from clinical infections identified in 3 dogs from New York and Minnesota.

**Figure 2:**
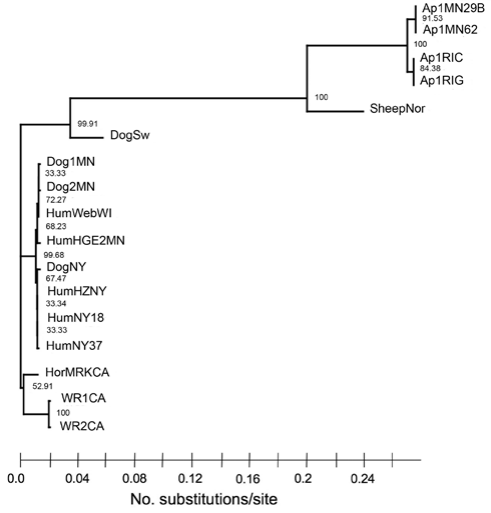
Maximum-likelihood phylogram of different variants of *Anaplasma phagocytophilum* based on the *p44ESup1/omp-1n* and intergenic region gene sequences created by using TREEFINDER (www.treefinder.de) with default values. The number of substitutions per site over 1,092 total sites is shown under the tree, and bootstrap support for each split (percentage of times recovered) is shown next to each branch of the tree.

When we performed a concatenated analysis of the *p44ESup1* and intergenic region *p44ESup1* to *msp2(p44)* using maximum-likelihood methods, we found strong support for 3 clades: a clade of strains from eastern North America; a clade of strains from western North America; and a clade comprising a sheep strain from Norway, a dog strain from Sweden, and the 4 *Ap-V1* strains from North America (Figure 2). Except for *Ap-V1*, the strains from eastern North America appeared to be closely related among themselves; the dog and human strains of *A. phagocytophilum* were indistinguishable from each other. Of note, a strain isolated from a dog in Sweden with clinical disease is on a separate branch from all US strains, including those from dogs in the United States.

The central hypervariable regions of *msp2/p44* and the flanking conserved sequences from 34 *Ap-V1* sequences were also aligned. The alignments showed the typical structure, including flanking LAKT residues and conserved framework residues such as C and WP described previously (*9*,*12*). Also, multiple hypervariable region variants were identified in each population of *A. phagocytophilum* (organisms characterized at a single time point from a single host). Some of the same variants were identified in different Rhode Island populations. No shared expression site variants were found between the Rhode Island and Minnesota *Ap-V1* strain sequences.

When comparing the *Ap-V1* expression site variants to genomic copies of the sequenced US human HZ strain, we found sequence identities >90% between 20/34 *Ap-V1* variants, including 100% identities of 5/34 *Ap-V1* variants. This level is comparable to that seen in most other US *A. phagocytophilum* strains. When compared with variants (non-HZ) identified directly from human infections, 10/34 *Ap-V1* variants were >90% identical. In contrast, none of the *Ap-V1* variants matched, with at least 70% identity, any previously identified MSP2/P44 expression site variants from strains from sheep in Norway. In general, little similarity was found between the *msp2/p44* hypervariable regions of US and European strain variants.

## Conclusions

Despite finding clear differences in the MSP2/P44 hypervariable region repertoire between US and European strains, we did not discover distinct repertoires in any US strains, including in *Ap-V1*. These findings agree with previous data that showed few differences by pulsed-field gel electrophoresis of 7 US strains (*13*) or by comparative microarray hybridization of 3 US strains (*14*). Our analysis focused on those hypervariable regions found frequently in the expression site. Because the genome repertoire contains ≈100 functional pseudogenes in each strain, complete genome sequencing may show differences in this repertoire not detected here.

The *p44ESup1*gene, upstream from *msp2/p44* on the same polycistronic mRNA transcript, gave the most phylogenetically useful information. This gene clearly distinguished *Ap-V1*from other US strains. Moreover, the resemblance of the *p44ESup1* gene in *Ap-V1* and in a strain from a sheep in Norway suggests that it may be a marker for a ruminant tropism of *A. phagocytophilum*. Also, phylogenetic trees based on the *p44ESup1* gene grouped *A. phagocytophilum* strains that cause clinical infections in US dogs or humans on the same branch. In fact, the genes from the 2 sources are indistinguishable, which may suggest a recent and common evolutionary origin of the US dog and human strains. Because these US data were obtained from a relatively small sampling of *A. phagocytophilum* infections (although from at least 2 states for the human, dog, and *Ap-V1* strains), these findings should be verified in a larger dataset.

The sequence divergence between strains in *p44ESup1* is similar to that in the downstream intergenic region. This intergenic region includes 2 divergent (54% and 58% identity in *Ap-V1*) binding sites for the transcription factor ApxR, which has been postulated to upregulate *msp2/p44* transcription in mammalian cells (*15*). Either the ApxR transcription factor has low specificity for sequence compared with secondary structure or it does not have the same biological mode of action in *Ap-V1* as in some other strains.

In summary, the *Ap-V1* expression site encoding *msp2/p44* was most similar to a strain from sheep in Norway. Strains causing clinical disease in humans and domestic dogs in the United States were indistinguishable.
